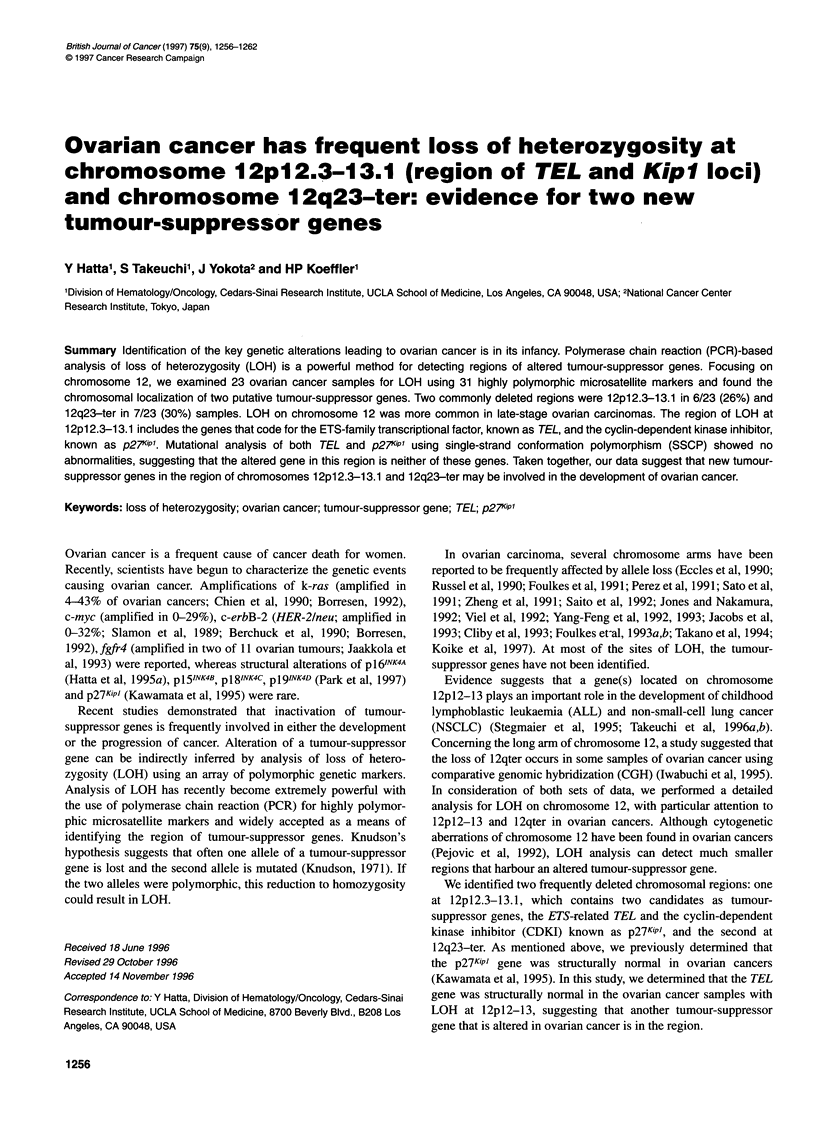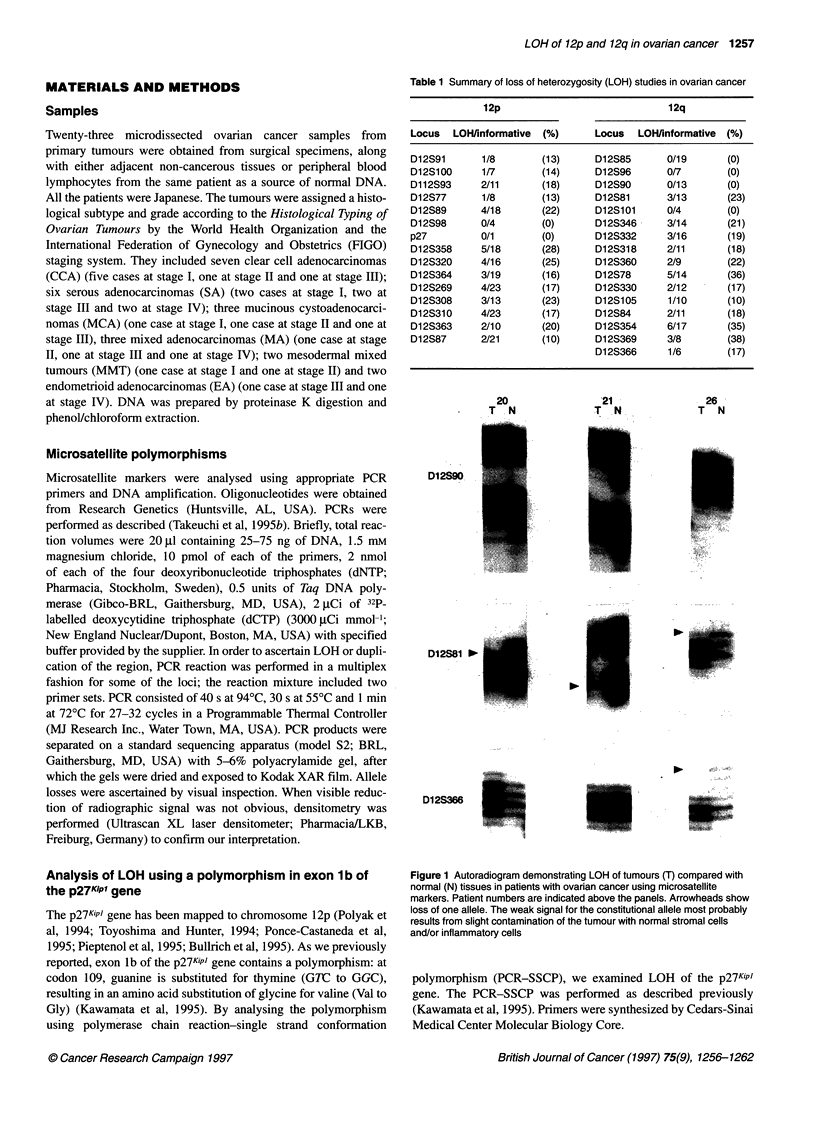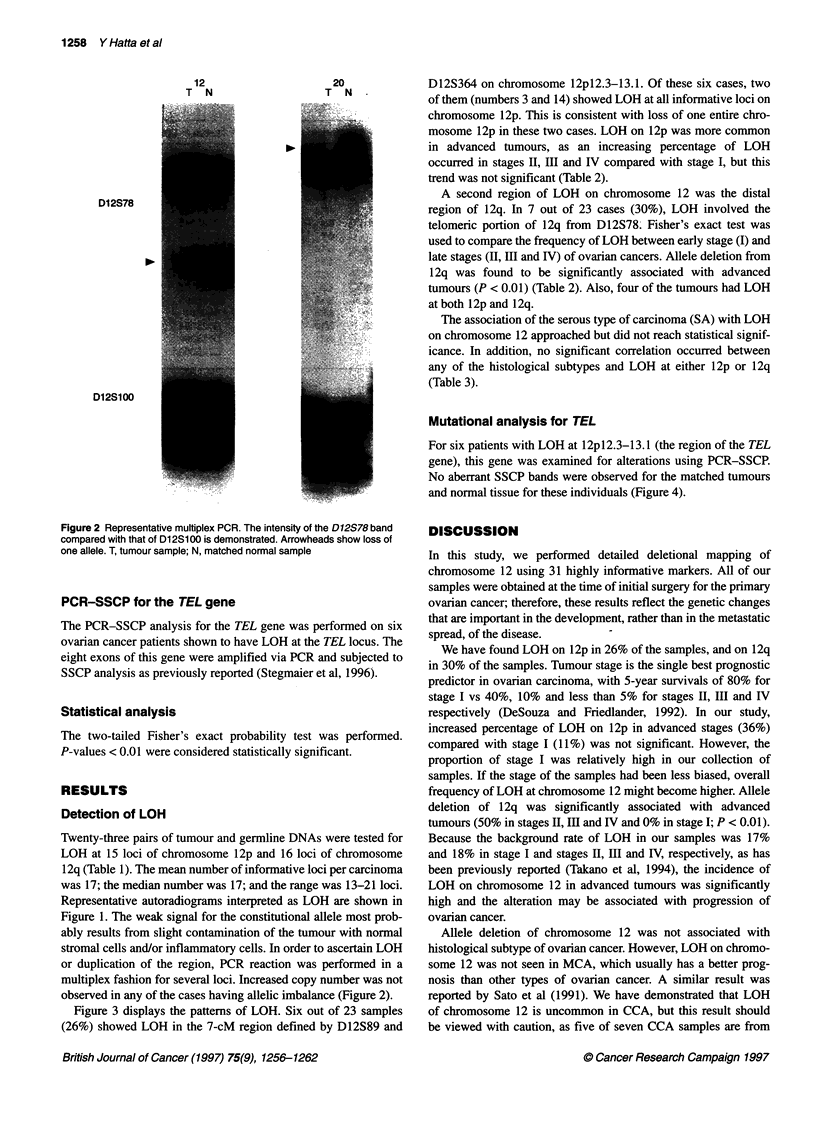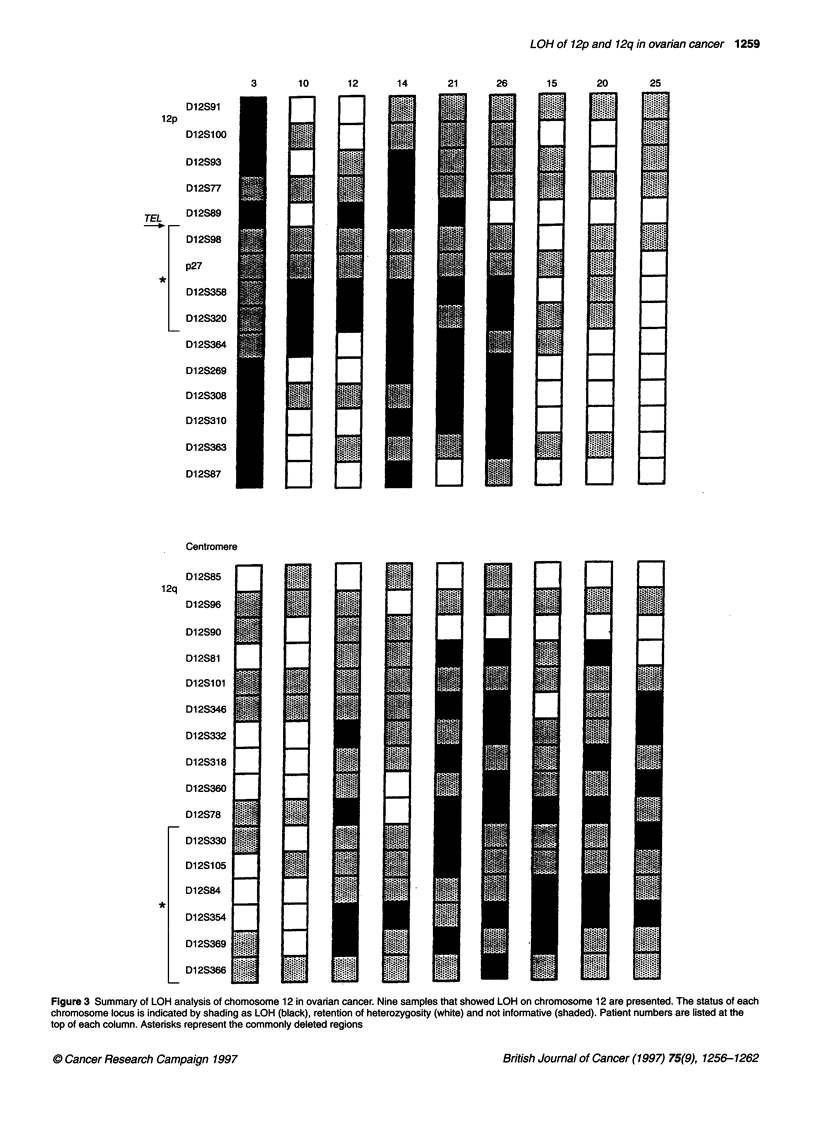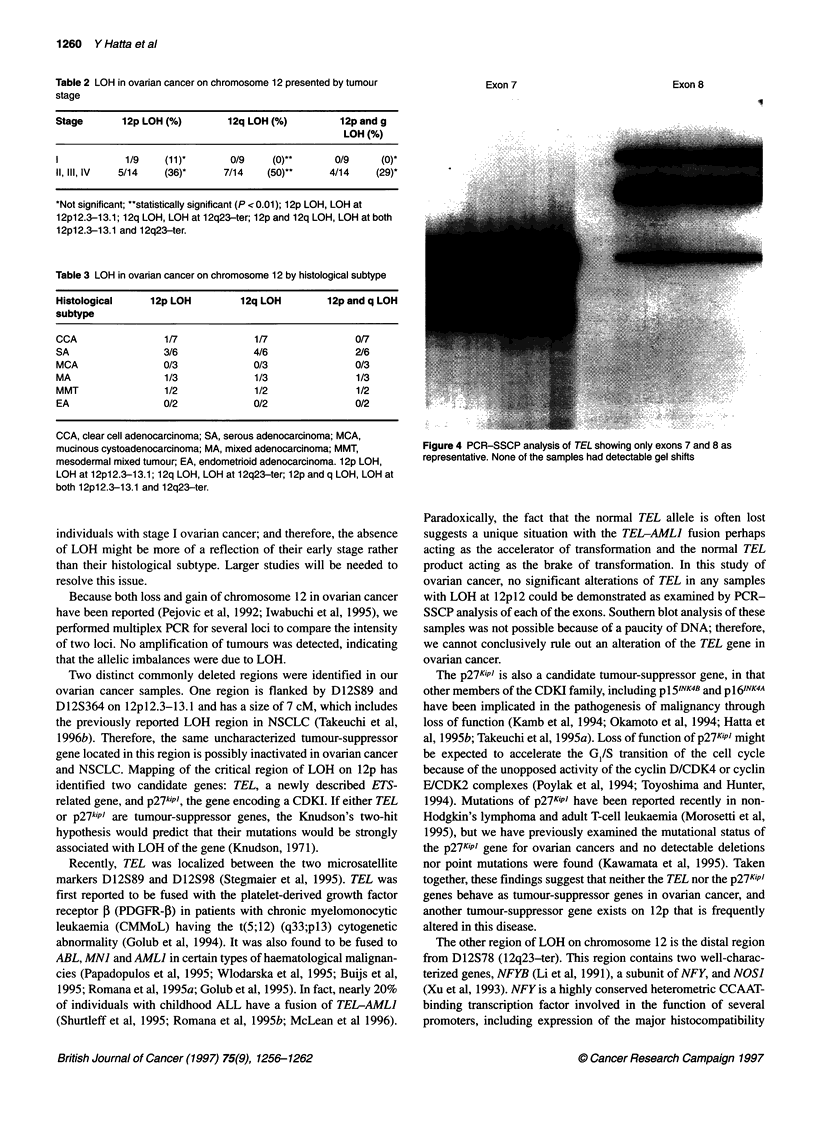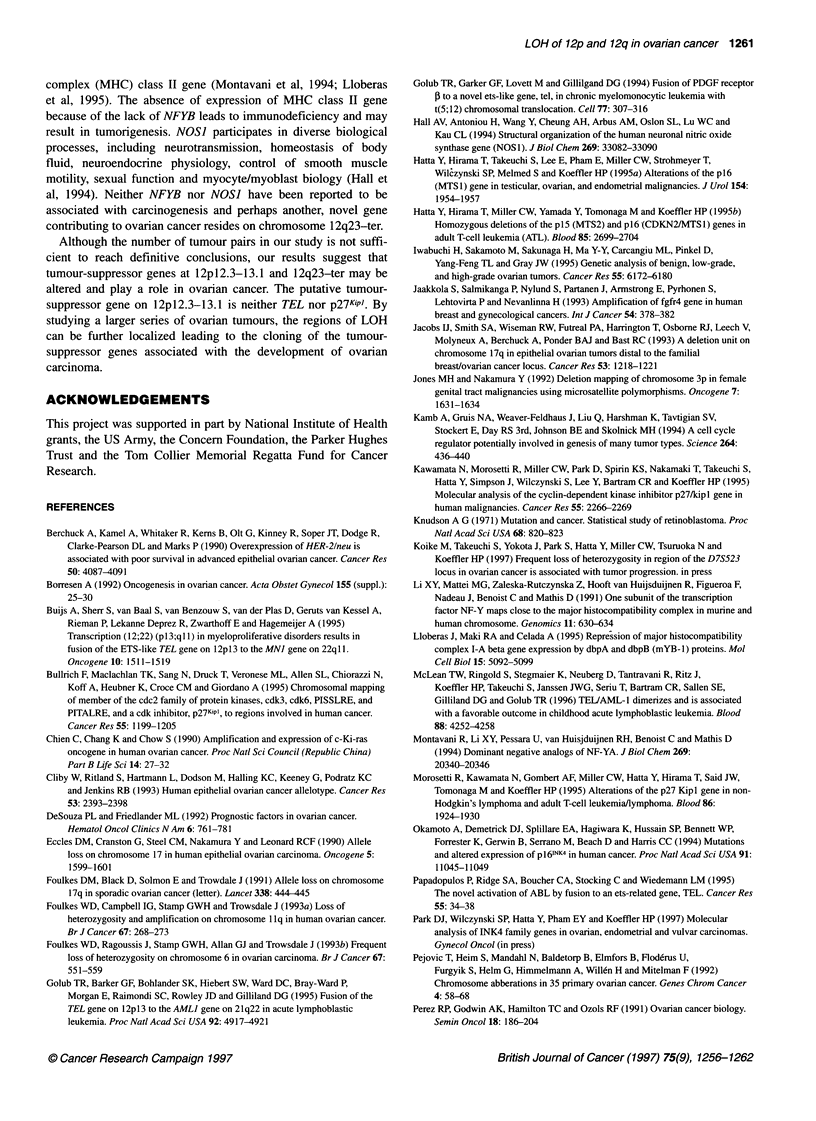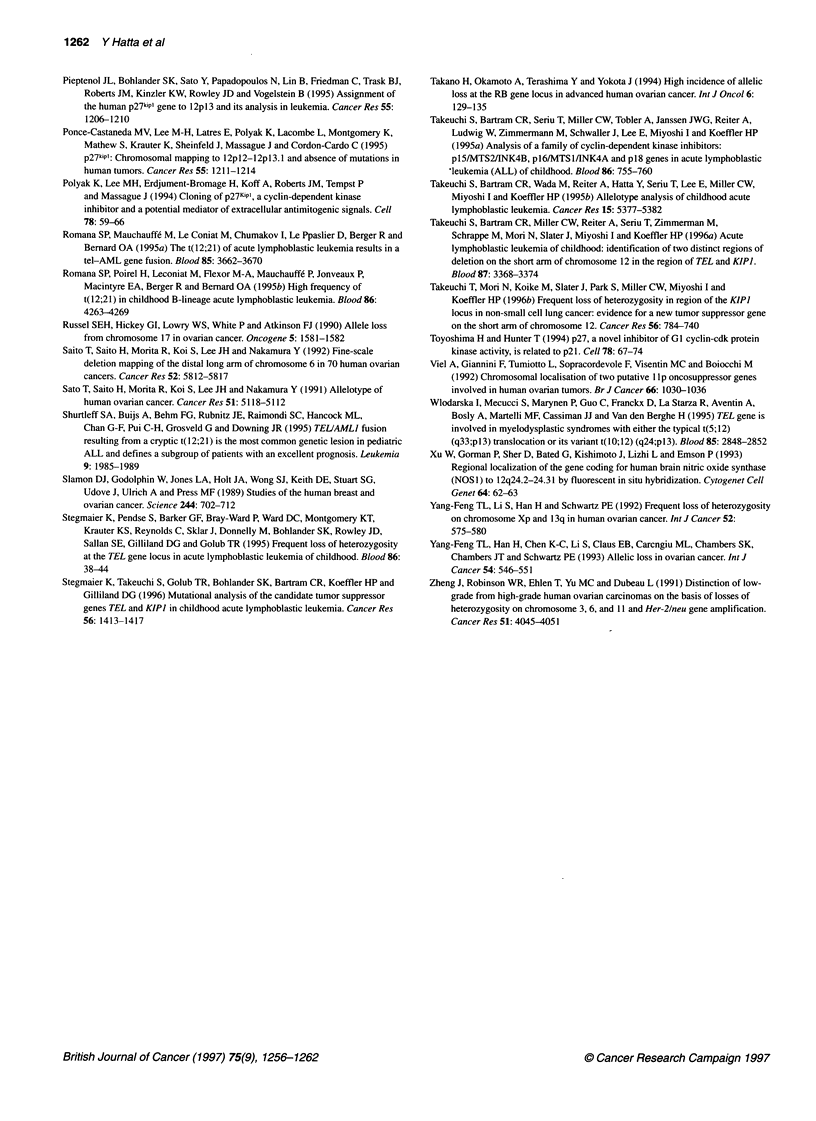# Ovarian cancer has frequent loss of heterozygosity at chromosome 12p12.3-13.1 (region of TEL and Kip1 loci) and chromosome 12q23-ter: evidence for two new tumour-suppressor genes.

**DOI:** 10.1038/bjc.1997.214

**Published:** 1997

**Authors:** Y. Hatta, S. Takeuchi, J. Yokota, H. P. Koeffler

**Affiliations:** Division of Hematology/Oncology, Cedars-Sinai Research Institute, UCLA School of Medicine, Los Angeles, CA 90048, USA.

## Abstract

**Images:**


British Journal of Cancer (1997) 75(9), 1256–1262
© 1997 Cancer Research Campaign

### Ovarian cancer has frequent loss of heterozygosity at chromosome 12p12.3–13.1 (region of *TEL* and *Kip1* loci) and chromosome 12q23–ter: evidence for two new tumour-suppressor genes

Y Hatta[1], S Takeuchi[1], J Yokota[2] and HP Koeffler[1]

[1]Division of Hematology/Oncology, Cedars-Sinai Research Institute, UCLA School of Medicine, Los Angeles, CA 90048, USA; [2]National Cancer Center Research Institute, Tokyo, Japan

**Summary** Identification of the key genetic alterations leading to ovarian cancer is in its infancy. Polymerase chain reaction (PCR)-based analysis of loss of heterozygosity (LOH) is a powerful method for detecting regions of altered tumour-suppressor genes. Focusing on chromosome 12, we examined 23 ovarian cancer samples for LOH using 31 highly polymorphic microsatellite markers and found the chromosomal localization of two putative tumour-suppressor genes. Two commonly deleted regions were 12p12.3–13.1 in 6/23 (26%) and 12q23–ter in 7/23 (30%) samples. LOH on chromosome 12 was more common in late-stage ovarian carcinomas. The region of LOH at 12p12.3–13.1 includes the genes that code for the ETS-family transcriptional factor, known as *TEL*, and the cyclin-dependent kinase inhibitor, known as *p27*$^{Kip1}$. Mutational analysis of both *TEL* and *p27*$^{Kip1}$ using single-strand conformation polymorphism (SSCP) showed no abnormalities, suggesting that the altered gene in this region is neither of these genes. Taken together, our data suggest that new tumour-suppressor genes in the region of chromosomes 12p12.3–13.1 and 12q23–ter may be involved in the development of ovarian cancer.

**Keywords:** loss of heterozygosity; ovarian cancer; tumour-suppressor gene; *TEL*; *p27*$^{Kip1}$

Ovarian cancer is a frequent cause of cancer death for women. Recently, scientists have begun to characterize the genetic events causing ovarian cancer. Amplifications of k-*ras* (amplified in 4–43% of ovarian cancers; Chien et al, 1990; Borresen, 1992), c-*myc* (amplified in 0–29%), c-*erb*B-2 (*HER-2/neu*; amplified in 0–32%; Slamon et al, 1989; Berchuck et al, 1990; Borresen, 1992), *fgfr4* (amplified in two of 11 ovarian tumours; Jaakkola et al, 1993) were reported, whereas structural alterations of p16$^{INK4A}$ (Hatta et al, 1995*a*), p15$^{INK4B}$, p18$^{INK4C}$, p19$^{INK4D}$ (Park et al, 1997) and p27$^{Kip1}$ (Kawamata et al, 1995) were rare.

Recent studies demonstrated that inactivation of tumour-suppressor genes is frequently involved in either the development or the progression of cancer. Alteration of a tumour-suppressor gene can be indirectly inferred by analysis of loss of heterozygosity (LOH) using an array of polymorphic genetic markers. Analysis of LOH has recently become extremely powerful with the use of polymerase chain reaction (PCR) for highly polymorphic microsatellite markers and widely accepted as a means of identifying the region of tumour-suppressor genes. Knudson's hypothesis suggests that often one allele of a tumour-suppressor gene is lost and the second allele is mutated (Knudson, 1971). If the two alleles were polymorphic, this reduction to homozygosity could result in LOH.

*Received 18 June 1996*
*Revised 29 October 1996*
*Accepted 14 November 1996*

*Correspondence to:* Y Hatta, Division of Hematology/Oncology, Cedars-Sinai Research Institute, UCLA School of Medicine, 8700 Beverly Blvd., B208 Los Angeles, CA 90048, USA

In ovarian carcinoma, several chromosome arms have been reported to be frequently affected by allele loss (Eccles et al, 1990; Russel et al, 1990; Foulkes et al, 1991; Perez et al, 1991; Sato et al, 1991; Zheng et al, 1991; Saito et al, 1992; Jones and Nakamura, 1992; Viel et al, 1992; Yang-Feng et al, 1992, 1993; Jacobs et al, 1993; Cliby et al, 1993; Foulkes et al, 1993*a,b*; Takano et al, 1994; Koike et al, 1997). At most of the sites of LOH, the tumour-suppressor genes have not been identified.

Evidence suggests that a gene(s) located on chromosome 12p12–13 plays an important role in the development of childhood lymphoblastic leukaemia (ALL) and non-small-cell lung cancer (NSCLC) (Stegmaier et al, 1995; Takeuchi et al, 1996*a,b*). Concerning the long arm of chromosome 12, a study suggested that the loss of 12qter occurs in some samples of ovarian cancer using comparative genomic hybridization (CGH) (Iwabuchi et al, 1995). In consideration of both sets of data, we performed a detailed analysis for LOH on chromosome 12, with particular attention to 12p12–13 and 12qter in ovarian cancers. Although cytogenetic aberrations of chromosome 12 have been found in ovarian cancers (Pejovic et al, 1992), LOH analysis can detect much smaller regions that harbour an altered tumour-suppressor gene.

We identified two frequently deleted chromosomal regions: one at 12p12.3–13.1, which contains two candidates as tumour-suppressor genes, the *ETS*-related *TEL* and the cyclin-dependent kinase inhibitor (CDKI) known as p27$^{Kip1}$, and the second at 12q23–ter. As mentioned above, we previously determined that the p27$^{Kip1}$ gene was structurally normal in ovarian cancers (Kawamata et al, 1995). In this study, we determined that the *TEL* gene was structurally normal in the ovarian cancer samples with LOH at 12p12–13, suggesting that another tumour-suppressor gene that is altered in ovarian cancer is in the region.

### MATERIALS AND METHODS

### Samples

Twenty-three microdissected ovarian cancer samples from primary tumours were obtained from surgical specimens, along with either adjacent non-cancerous tissues or peripheral blood lymphocytes from the same patient as a source of normal DNA. All the patients were Japanese. The tumours were assigned a histological subtype and grade according to the *Histological Typing of Ovarian Tumours* by the World Health Organization and the International Federation of Gynecology and Obstetrics (FIGO) staging system. They included seven clear cell adenocarcinomas (CCA) (five cases at stage I, one at stage II and one at stage III); six serous adenocarcinomas (SA) (two cases at stage I, two at stage III and two at stage IV); three mucinous cystoadenocarcinomas (MCA) (one case at stage I, one case at stage II and one at stage III), three mixed adenocarcinomas (MA) (one case at stage II, one at stage III and one at stage IV); two mesodermal mixed tumours (MMT) (one case at stage I and one at stage II) and two endometrioid adenocarcinomas (EA) (one case at stage III and one at stage IV). DNA was prepared by proteinase K digestion and phenol/chloroform extraction.

### Microsatellite polymorphisms

Microsatellite markers were analysed using appropriate PCR primers and DNA amplification. Oligonucleotides were obtained from Research Genetics (Huntsville, AL, USA). PCRs were performed as described (Takeuchi et al, 1995*b*). Briefly, total reaction volumes were 20 μl containing 25–75 ng of DNA, 1.5 mM magnesium chloride, 10 pmol of each of the primers, 2 nmol of each of the four deoxyribonucleotide triphosphates (dNTP; Pharmacia, Stockholm, Sweden), 0.5 units of *Taq* DNA polymerase (Gibco-BRL, Gaithersburg, MD, USA), 2 μCi of $^{32}$P-labelled deoxycytidine triphosphate (dCTP) (3000 μCi mmol$^{-1}$; New England Nuclear/Dupont, Boston, MA, USA) with specified buffer provided by the supplier. In order to ascertain LOH or duplication of the region, PCR reaction was performed in a multiplex fashion for some of the loci; the reaction mixture included two primer sets. PCR consisted of 40 s at 94°C, 30 s at 55°C and 1 min at 72°C for 27–32 cycles in a Programmable Thermal Controller (MJ Research Inc., Water Town, MA, USA). PCR products were separated on a standard sequencing apparatus (model S2; BRL, Gaithersburg, MD, USA) with 5–6% polyacrylamide gel, after which the gels were dried and exposed to Kodak XAR film. Allele losses were ascertained by visual inspection. When visible reduction of radiographic signal was not obvious, densitometry was performed (Ultrascan XL laser densitometer; Pharmacia/LKB, Freiburg, Germany) to confirm our interpretation.

### Analysis of LOH using a polymorphism in exon 1b of the p27$^{Kip1}$ gene

The p27$^{Kip1}$ gene has been mapped to chromosome 12p (Polyak et al, 1994; Toyoshima and Hunter, 1994; Ponce-Castaneda et al, 1995; Pieptenol et al, 1995; Bullrich et al, 1995). As we previously reported, exon 1b of the p27$^{Kip1}$ gene contains a polymorphism: at codon 109, guanine is substituted for thymine (GTC to GGC), resulting in an amino acid substitution of glycine for valine (Val to Gly) (Kawamata et al, 1995). By analysing the polymorphism using polymerase chain reaction–single strand conformation polymorphism (PCR–SSCP), we examined LOH of the p27$^{Kip1}$ gene. The PCR–SSCP was performed as described previously (Kawamata et al, 1995). Primers were synthesized by Cedars-Sinai Medical Center Molecular Biology Core.

**Table 1** Summary of loss of heterozygosity (LOH) studies in ovarian cancer

| 12p | | | 12q | | |
|---|---|---|---|---|---|
| Locus | LOH/informative | (%) | Locus | LOH/informative | (%) |
| D12S91 | 1/8 | (13) | D12S85 | 0/19 | (0) |
| D12S100 | 1/7 | (14) | D12S96 | 0/7 | (0) |
| D112S93 | 2/11 | (18) | D12S90 | 0/13 | (0) |
| D12S77 | 1/8 | (13) | D12S81 | 3/13 | (23) |
| D12S89 | 4/18 | (22) | D12S101 | 0/4 | (0) |
| D12S98 | 0/4 | (0) | D12S346 | 3/14 | (21) |
| p27 | 0/1 | (0) | D12S332 | 3/16 | (19) |
| D12S358 | 5/18 | (28) | D12S318 | 2/11 | (18) |
| D12S320 | 4/16 | (25) | D12S360 | 2/9 | (22) |
| D12S364 | 3/19 | (16) | D12S78 | 5/14 | (36) |
| D12S269 | 4/23 | (17) | D12S330 | 2/12 | (17) |
| D12S308 | 3/13 | (23) | D12S105 | 1/10 | (10) |
| D12S310 | 4/23 | (17) | D12S84 | 2/11 | (18) |
| D12S363 | 2/10 | (20) | D12S354 | 6/17 | (35) |
| D12S87 | 2/21 | (10) | D12S369 | 3/8 | (38) |
| | | | D12S366 | 1/6 | (17) |

**Figure 1** Autoradiogram demonstrating LOH of tumours (T) compared with normal (N) tissues in patients with ovarian cancer using microsatellite markers. Patient numbers are indicated above the panels. Arrowheads show loss of one allele. The weak signal for the constitutional allele most probably results from slight contamination of the tumour with normal stromal cells and/or inflammatory cells

© Cancer Research Campaign 1997

**Figure 2** Representative multiplex PCR. The intensity of the *D12S78* band compared with that of D12S100 is demonstrated. Arrowheads show loss of one allele. T, tumour sample; N, matched normal sample

### PCR–SSCP for the *TEL* gene

The PCR–SSCP analysis for the *TEL* gene was performed on six ovarian cancer patients shown to have LOH at the *TEL* locus. The eight exons of this gene were amplified via PCR and subjected to SSCP analysis as previously reported (Stegmaier et al, 1996).

### Statistical analysis

The two-tailed Fisher's exact probability test was performed. *P*-values < 0.01 were considered statistically significant.

### RESULTS

### Detection of LOH

Twenty-three pairs of tumour and germline DNAs were tested for LOH at 15 loci of chromosome 12p and 16 loci of chromosome 12q (Table 1). The mean number of informative loci per carcinoma was 17; the median number was 17; and the range was 13–21 loci. Representative autoradiograms interpreted as LOH are shown in Figure 1. The weak signal for the constitutional allele most probably results from slight contamination of the tumour with normal stromal cells and/or inflammatory cells. In order to ascertain LOH or duplication of the region, PCR reaction was performed in a multiplex fashion for several loci. Increased copy number was not observed in any of the cases having allelic imbalance (Figure 2).

Figure 3 displays the patterns of LOH. Six out of 23 samples (26%) showed LOH in the 7-cM region defined by D12S89 and D12S364 on chromosome 12p12.3–13.1. Of these six cases, two of them (numbers 3 and 14) showed LOH at all informative loci on chromosome 12p. This is consistent with loss of one entire chromosome 12p in these two cases. LOH on 12p was more common in advanced tumours, as an increasing percentage of LOH occurred in stages II, III and IV compared with stage I, but this trend was not significant (Table 2).

A second region of LOH on chromosome 12 was the distal region of 12q. In 7 out of 23 cases (30%), LOH involved the telomeric portion of 12q from D12S78. Fisher's exact test was used to compare the frequency of LOH between early stage (I) and late stages (II, III and IV) of ovarian cancers. Allele deletion from 12q was found to be significantly associated with advanced tumours ($P < 0.01$) (Table 2). Also, four of the tumours had LOH at both 12p and 12q.

The association of the serous type of carcinoma (SA) with LOH on chromosome 12 approached but did not reach statistical significance. In addition, no significant correlation occurred between any of the histological subtypes and LOH at either 12p or 12q (Table 3).

### Mutational analysis for *TEL*

For six patients with LOH at 12p12.3–13.1 (the region of the *TEL* gene), this gene was examined for alterations using PCR–SSCP. No aberrant SSCP bands were observed for the matched tumours and normal tissue for these individuals (Figure 4).

### DISCUSSION

In this study, we performed detailed deletional mapping of chromosome 12 using 31 highly informative markers. All of our samples were obtained at the time of initial surgery for the primary ovarian cancer; therefore, these results reflect the genetic changes that are important in the development, rather than in the metastatic spread, of the disease.

We have found LOH on 12p in 26% of the samples, and on 12q in 30% of the samples. Tumour stage is the single best prognostic predictor in ovarian carcinoma, with 5-year survivals of 80% for stage I vs 40%, 10% and less than 5% for stages II, III and IV respectively (DeSouza and Friedlander, 1992). In our study, increased percentage of LOH on 12p in advanced stages (36%) compared with stage I (11%) was not significant. However, the proportion of stage I was relatively high in our collection of samples. If the stage of the samples had been less biased, overall frequency of LOH at chromosome 12 might become higher. Allele deletion of 12q was significantly associated with advanced tumours (50% in stages II, III and IV and 0% in stage I; $P < 0.01$). Because the background rate of LOH in our samples was 17% and 18% in stage I and stages II, III and IV, respectively, as has been previously reported (Takano et al, 1994), the incidence of LOH on chromosome 12 in advanced tumours was significantly high and the alteration may be associated with progression of ovarian cancer.

Allele deletion of chromosome 12 was not associated with histological subtype of ovarian cancer. However, LOH on chromosome 12 was not seen in MCA, which usually has a better prognosis than other types of ovarian cancer. A similar result was reported by Sato et al (1991). We have demonstrated that LOH of chromosome 12 is uncommon in CCA, but this result should be viewed with caution, as five of seven CCA samples are from

© Cancer Research Campaign 1997

**Figure 3** Summary of LOH analysis of chomosome 12 in ovarian cancer. Nine samples that showed LOH on chromosome 12 are presented. The status of each chromosome locus is indicated by shading as LOH (black), retention of heterozygosity (white) and not informative (shaded). Patient numbers are listed at the top of each column. Asterisks represent the commonly deleted regions

**Table 2** LOH in ovarian cancer on chromosome 12 presented by tumour stage

| Stage | 12p LOH (%) | | 12q LOH (%) | | 12p and g LOH (%) | |
|---|---|---|---|---|---|---|
| I | 1/9 | (11)* | 0/9 | (0)** | 0/9 | (0)* |
| II, III, IV | 5/14 | (36)* | 7/14 | (50)** | 4/14 | (29)* |

*Not significant; **statistically significant ($P < 0.01$); 12p LOH, LOH at 12p12.3–13.1; 12q LOH, LOH at 12q23–ter; 12p and 12q LOH, LOH at both 12p12.3–13.1 and 12q23–ter.

**Table 3** LOH in ovarian cancer on chromosome 12 by histological subtype

| Histological subtype | 12p LOH | 12q LOH | 12p and q LOH |
|---|---|---|---|
| CCA | 1/7 | 1/7 | 0/7 |
| SA | 3/6 | 4/6 | 2/6 |
| MCA | 0/3 | 0/3 | 0/3 |
| MA | 1/3 | 1/3 | 1/3 |
| MMT | 1/2 | 1/2 | 1/2 |
| EA | 0/2 | 0/2 | 0/2 |

CCA, clear cell adenocarcinoma; SA, serous adenocarcinoma; MCA, mucinous cystoadenocarcinoma; MA, mixed adenocarcinoma; MMT, mesodermal mixed tumour; EA, endometrioid adenocarcinoma. 12p LOH, LOH at 12p12.3–13.1; 12q LOH, LOH at 12q23–ter; 12p and q LOH, LOH at both 12p12.3–13.1 and 12q23–ter.

**Figure 4** PCR–SSCP analysis of *TEL* showing only exons 7 and 8 as representative. None of the samples had detectable gel shifts individuals with stage I ovarian cancer; and therefore, the absence of LOH might be more of a reflection of their early stage rather than their histological subtype. Larger studies will be needed to resolve this issue.

Because both loss and gain of chromosome 12 in ovarian cancer have been reported (Pejovic et al, 1992; Iwabuchi et al, 1995), we performed multiplex PCR for several loci to compare the intensity of two loci. No amplification of tumours was detected, indicating that the allelic imbalances were due to LOH.

Two distinct commonly deleted regions were identified in our ovarian cancer samples. One region is flanked by D12S89 and D12S364 on 12p12.3–13.1 and has a size of 7 cM, which includes the previously reported LOH region in NSCLC (Takeuchi et al, 1996*b*). Therefore, the same uncharacterized tumour-suppressor gene located in this region is possibly inactivated in ovarian cancer and NSCLC. Mapping of the critical region of LOH on 12p has identified two candidate genes: *TEL*, a newly described *ETS*-related gene, and $p27^{kip1}$, the gene encoding a CDKI. If either *TEL* or $p27^{kip1}$ are tumour-suppressor genes, the Knudson's two-hit hypothesis would predict that their mutations would be strongly associated with LOH of the gene (Knudson, 1971).

Recently, *TEL* was localized between the two microsatellite markers D12S89 and D12S98 (Stegmaier et al, 1995). *TEL* was first reported to be fused with the platelet-derived growth factor receptor β (PDGFR-β) in patients with chronic myelomonocytic leukaemia (CMMoL) having the t(5;12) (q33;p13) cytogenetic abnormality (Golub et al, 1994). It was also found to be fused to *ABL*, *MN1* and *AML1* in certain types of haematological malignancies (Papadopulos et al, 1995; Wlodarska et al, 1995; Buijs et al, 1995; Romana et al, 1995*a*; Golub et al, 1995). In fact, nearly 20% of individuals with childhood ALL have a fusion of *TEL–AML1* (Shurtleff et al, 1995; Romana et al, 1995*b*; McLean et al 1996). Paradoxically, the fact that the normal *TEL* allele is often lost suggests a unique situation with the *TEL–AML1* fusion perhaps acting as the accelerator of transformation and the normal *TEL* product acting as the brake of transformation. In this study of ovarian cancer, no significant alterations of *TEL* in any samples with LOH at 12p12 could be demonstrated as examined by PCR–SSCP analysis of each of the exons. Southern blot analysis of these samples was not possible because of a paucity of DNA; therefore, we cannot conclusively rule out an alteration of the *TEL* gene in ovarian cancer.

The $p27^{Kip1}$ is also a candidate tumour-suppressor gene, in that other members of the CDKI family, including $p15^{INK4B}$ and $p16^{INK4A}$ have been implicated in the pathogenesis of malignancy through loss of function (Kamb et al, 1994; Okamoto et al, 1994; Hatta et al, 1995*b*; Takeuchi et al, 1995*a*). Loss of function of $p27^{Kip1}$ might be expected to accelerate the $G_1$/S transition of the cell cycle because of the unopposed activity of the cyclin D/CDK4 or cyclin E/CDK2 complexes (Poylak et al, 1994; Toyoshima and Hunter, 1994). Mutations of $p27^{Kip1}$ have been reported recently in non-Hodgkin's lymphoma and adult T-cell leukaemia (Morosetti et al, 1995), but we have previously examined the mutational status of the $p27^{Kip1}$ gene for ovarian cancers and no detectable deletions nor point mutations were found (Kawamata et al, 1995). Taken together, these findings suggest that neither the *TEL* nor the $p27^{Kip1}$ genes behave as tumour-suppressor genes in ovarian cancer, and another tumour-suppressor gene exists on 12p that is frequently altered in this disease.

The other region of LOH on chromosome 12 is the distal region from D12S78 (12q23–ter). This region contains two well-characterized genes, *NFYB* (Li et al, 1991), a subunit of *NFY*, and *NOS1* (Xu et al, 1993). *NFY* is a highly conserved heterometric CCAAT-binding transcription factor involved in the function of several promoters, including expression of the major histocompatibility

 *© Cancer Research Campaign 1997*

complex (MHC) class II gene (Montavani et al, 1994; Lloberas et al, 1995). The absence of expression of MHC class II gene because of the lack of *NFYB* leads to immunodeficiency and may result in tumorigenesis. *NOS1* participates in diverse biological processes, including neurotransmission, homeostasis of body fluid, neuroendocrine physiology, control of smooth muscle motility, sexual function and myocyte/myoblast biology (Hall et al, 1994). Neither *NFYB* nor *NOS1* have been reported to be associated with carcinogenesis and perhaps another, novel gene contributing to ovarian cancer resides on chromosome 12q23–ter.

Although the number of tumour pairs in our study is not sufficient to reach definitive conclusions, our results suggest that tumour-suppressor genes at 12p12.3–13.1 and 12q23–ter may be altered and play a role in ovarian cancer. The putative tumour-suppressor gene on 12p12.3–13.1 is neither *TEL* nor $p27^{Kip1}$. By studying a larger series of ovarian tumours, the regions of LOH can be further localized leading to the cloning of the tumour-suppressor genes associated with the development of ovarian carcinoma.

### ACKNOWLEDGEMENTS

This project was supported in part by National Institute of Health grants, the US Army, the Concern Foundation, the Parker Hughes Trust and the Tom Collier Memorial Regatta Fund for Cancer Research.

### REFERENCES

Berchuck A, Kamel A, Whitaker R, Kerns B, Olt G, Kinney R, Soper JT, Dodge R, Clarke-Pearson DL and Marks P (1990) Overexpression of *HER-2/neu* is associated with poor survival in advanced epithelial ovarian cancer. *Cancer Res* **50**: 4087–4091

Borresen A (1992) Oncogenesis in ovarian cancer. *Acta Obstet Gynecol* **155** (suppl.): 25–30

Buijs A, Sherr S, van Baal S, van Benzouw S, van der Plas D, Geruts van Kessel A, Rieman P, Lekanne Deprez R, Zwarthoff E and Hagemeijer A (1995) Transcription (12;22) (p13;q11) in myeloproliferative disorders results in fusion of the ETS-like *TEL* gene on 12p13 to the *MN1* gene on 22q11. *Oncogene* **10**: 1511–1519

Bullrich F, Maclachlan TK, Sang N, Druck T, Veronese ML, Allen SL, Chiorazzi N, Koff A, Heubner K, Croce CM and Giordano A (1995) Chromosomal mapping of member of the cdc2 family of protein kinases, cdk3, cdk6, PISSLRE, and PITALRE, and a cdk inhibitor, $p27^{Kip1}$, to regions involved in human cancer. *Cancer Res* **55**: 1199–1205

Chien C, Chang K and Chow S (1990) Amplification and expression of c-Ki-ras oncogene in human ovarian cancer. *Proc Natl Sci Council (Republic China) Part B Life Sci* **14**: 27–32

Cliby W, Ritland S, Hartmann L, Dodson M, Halling KC, Keeney G, Podratz KC and Jenkins RB (1993) Human epithelial ovarian cancer allelotype. *Cancer Res* **53**: 2393–2398

DeSouza PL and Friedlander ML (1992) Prognostic factors in ovarian cancer. *Hematol Oncol Clinics N Am* **6**: 761–781

Eccles DM, Cranston G, Steel CM, Nakamura Y and Leonard RCF (1990) Allele loss on chromosome 17 in human epithelial ovarian carcinoma. *Oncogene* **5**: 1599–1601

Foulkes DM, Black D, Solmon E and Trowdale J (1991) Allele loss on chromosome 17q in sporadic ovarian cancer (letter). *Lancet* **338**: 444–445

Foulkes WD, Campbell IG, Stamp GWH and Trowsdale J (1993*a*) Loss of heterozygosity and amplification on chromosome 11q in human ovarian cancer. *Br J Cancer* **67**: 268–273

Foulkes WD, Ragoussis J, Stamp GWH, Allan GJ and Trowsdale J (1993*b*) Frequent loss of heterozygosity on chromosome 6 in ovarian carcinoma. *Br J Cancer* **67**: 551–559

Golub TR, Barker GF, Bohlander SK, Hiebert SW, Ward DC, Bray-Ward P, Morgan E, Raimondi SC, Rowley JD and Gilliland DG (1995) Fusion of the *TEL* gene on 12p13 to the *AML1* gene on 21q22 in acute lymphoblastic leukemia. *Proc Natl Acad Sci USA* **92**: 4917–4921

Golub TR, Garker GF, Lovett M and Gillilgand DG (1994) Fusion of PDGF receptor β to a novel ets-like gene, tel, in chronic myelomonocytic leukemia with t(5;12) chromosomal translocation. *Cell* **77**: 307–316

Hall AV, Antoniou H, Wang Y, Cheung AH, Arbus AM, Oslon SL, Lu WC and Kau CL (1994) Structural organization of the human neuronal nitric oxide synthase gene (NOS1). *J Biol Chem* **269**: 33082–33090

Hatta Y, Hirama T, Takeuchi S, Lee E, Pham E, Miller CW, Strohmeyer T, Wilčzynski SP, Melmed S and Koeffler HP (1995*a*) Alterations of the p16 (MTS1) gene in testicular, ovarian, and endometrial malignancies. *J Urol* **154**: 1954–1957

Hatta Y, Hirama T, Miller CW, Yamada Y, Tomonaga M and Koeffler HP (1995*b*) Homozygous deletions of the p15 (MTS2) and p16 (CDKN2/MTS1) genes in adult T-cell leukemia (ATL). *Blood* **85**: 2699–2704

Iwabuchi H, Sakamoto M, Sakunaga H, Ma Y-Y, Carcangiu ML, Pinkel D, Yang-Feng TL and Gray JW (1995) Genetic analysis of benign, low-grade, and high-grade ovarian tumors. *Cancer Res* **55**: 6172–6180

Jaakkola S, Salmikanga P, Nylund S, Partanen J, Armstrong E, Pyrhonen S, Lehtovirta P and Nevanlinna H (1993) Amplification of fgfr4 gene in human breast and gynecological cancers. *Int J Cancer* **54**: 378–382

Jacobs IJ, Smith SA, Wiseman RW, Futreal PA, Harrington T, Osborne RJ, Leech V, Molyneux A, Berchuck A, Ponder BAJ and Bast RC (1993) A deletion unit on chromosome 17q in epithelial ovarian tumors distal to the familial breast/ovarian cancer locus. *Cancer Res* **53**: 1218–1221

Jones MH and Nakamura Y (1992) Deletion mapping of chromosome 3p in female genital tract malignancies using microsatellite polymorphisms. *Oncogene* **7**: 1631–1634

Kamb A, Gruis NA, Weaver-Feldhaus J, Liu Q, Harshman K, Tavtigian SV, Stockert E, Day RS 3rd, Johnson BE and Skolnick MH (1994) A cell cycle regulator potentially involved in genesis of many tumor types. *Science* **264**: 436–440

Kawamata N, Morosetti R, Miller CW, Park D, Spirin KS, Nakamaki T, Takeuchi S, Hatta Y, Simpson J, Wilczynski S, Lee Y, Bartram CR and Koeffler HP (1995) Molecular analysis of the cyclin-dependent kinase inhibitor p27/kip1 gene in human malignancies. *Cancer Res* **55**: 2266–2269

Knudson A G (1971) Mutation and cancer. Statistical study of retinoblastoma. *Proc Natl Acad Sci USA* **68**: 820–823

Koike M, Takeuchi S, Yokota J, Park S, Hatta Y, Miller CW, Tsuruoka N and Koeffler HP (1997) Frequent loss of heterozygosity in region of the *D7S523* locus in ovarian cancer is associated with tumor progression. in press

Li XY, Mattei MG, Zaleska-Rutczynska Z, Hooft van Huijsduijnen R, Figueroa F, Nadeau J, Benoist C and Mathis D (1991) One subunit of the transcription factor NF-Y maps close to the major histocompatibility complex in murine and human chromosome. *Genomics* **11**: 630–634

Lloberas J, Maki RA and Celada A (1995) Repression of major histocompatibility complex I-A beta gene expression by dbpA and dbpB (mYB-1) proteins. *Mol Cell Biol* **15**: 5092–5099

McLean TW, Ringold S, Stegmaier K, Neuberg D, Tantravani R, Ritz J, Koeffler HP, Takeuchi S, Janssen JWG, Seriu T, Bartram CR, Sallen SE, Gilliland DG and Golub TR (1996) TEL/AML-1 dimerizes and is associated with a favorable outcome in childhood acute lymphoblastic leukemia. *Blood* **88**: 4252–4258

Montavani R, Li XY, Pessara U, van Huisjduijnen RH, Benoist C and Mathis D (1994) Dominant negative analogs of NF-YA. *J Biol Chem* **269**: 20340–20346

Morosetti R, Kawamata N, Gombert AF, Miller CW, Hatta Y, Hirama T, Said JW, Tomonaga M and Koeffler HP (1995) Alterations of the p27 Kip1 gene in non-Hodgkin's lymphoma and adult T-cell leukemia/lymphoma. *Blood* **86**: 1924–1930

Okamoto A, Demetrick DJ, Splillare EA, Hagiwara K, Hussain SP, Bennett WP, Forrester K, Gerwin B, Serrano M, Beach D and Harris CC (1994) Mutations and altered expression of $p16^{INK4}$ in human cancer. *Proc Natl Acad Sci USA* **91**: 11045–11049

Papadopulos P, Ridge SA, Boucher CA, Stocking C and Wiedemann LM (1995) The novel activation of ABL by fusion to an ets-related gene, TEL. *Cancer Res* **55**: 34–38

Park DJ, Wilczynski SP, Hatta Y, Pham EY and Koeffler HP (1997) Molecular analysis of INK4 family genes in ovarian, endometrial and vulvar carcinomas. *Gynecol Oncol* (in press)

Pejovic T, Heim S, Mandahl N, Baldetorp B, Elmfors B, Flodérus U, Furgyik S, Helm G, Himmelmann A, Willén H and Mitelman F (1992) Chromosome abberations in 35 primary ovarian cancer. *Genes Chrom Cancer* **4**: 58–68

Perez RP, Godwin AK, Hamilton TC and Ozols RF (1991) Ovarian cancer biology. *Semin Oncol* **18**: 186–204

© Cancer Research Campaign 1997

Pieptenol JL, Bohlander SK, Sato Y, Papadopoulos N, Lin B, Friedman C, Trask BJ, Roberts JM, Kinzler KW, Rowley JD and Vogelstein B (1995) Assignment of the human p27$^{kip1}$ gene to 12p13 and its analysis in leukemia. *Cancer Res* **55**: 1206–1210

Ponce-Castaneda MV, Lee M-H, Latres E, Polyak K, Lacombe L, Montgomery K, Mathew S, Krauter K, Sheinfeld J, Massague J and Cordon-Cardo C (1995) p27$^{kip1}$: Chromosomal mapping to 12p12–12p13.1 and absence of mutations in human tumors. *Cancer Res* **55**: 1211–1214

Polyak K, Lee MH, Erdjument-Bromage H, Koff A, Roberts JM, Tempst P and Massague J (1994) Cloning of p27$^{Kip1}$, a cyclin-dependent kinase inhibitor and a potential mediator of extracellular antimitogenic signals. *Cell* **78**: 59–66

Romana SP, Mauchauffé M, Le Coniat M, Chumakov I, Le Ppaslier D, Berger R and Bernard OA (1995*a*) The t(12;21) of acute lymphoblastic leukemia results in a tel–AML gene fusion. *Blood* **85**: 3662–3670

Romana SP, Poirel H, Leconiat M, Flexor M-A, Mauchauffé P, Jonveaux P, Macintyre EA, Berger R and Bernard OA (1995*b*) High frequency of t(12;21) in childhood B-lineage acute lymphoblastic leukemia. *Blood* **86**: 4263–4269

Russel SEH, Hickey GI, Lowry WS, White P and Atkinson FJ (1990) Allele loss from chromosome 17 in ovarian cancer. *Oncogene* **5**: 1581–1582

Saito T, Saito H, Morita R, Koi S, Lee JH and Nakamura Y (1992) Fine-scale deletion mapping of the distal long arm of chromosome 6 in 70 human ovarian cancers. *Cancer Res* **52**: 5812–5817

Sato T, Saito H, Morita R, Koi S, Lee JH and Nakamura Y (1991) Allelotype of human ovarian cancer. *Cancer Res* **51**: 5118–5112

Shurtleff SA, Buijs A, Behm FG, Rubnitz JE, Raimondi SC, Hancock ML, Chan G-F, Pui C-H, Grosveld G and Downing JR (1995) *TEL/AML1* fusion resulting from a cryptic t(12;21) is the most common genetic lesion in pediatric ALL and defines a subgroup of patients with an excellent prognosis. *Leukemia* **9**: 1985–1989

Slamon DJ, Godolphin W, Jones LA, Holt JA, Wong SJ, Keith DE, Stuart SG, Udove J, Ulrich A and Press MF (1989) Studies of the human breast and ovarian cancer. *Science* **244**: 702–712

Stegmaier K, Pendse S, Barker GF, Bray-Ward P, Ward DC, Montgomery KT, Krauter KS, Reynolds C, Sklar J, Donnelly M, Bohlander SK, Rowley JD, Sallan SE, Gilliland DG and Golub TR (1995) Frequent loss of heterozygosity at the *TEL* gene locus in acute lymphoblastic leukemia of childhood. *Blood* **86**: 38–44

Stegmaier K, Takeuchi S, Golub TR, Bohlander SK, Bartram CR, Koeffler HP and Gilliland DG (1996) Mutational analysis of the candidate tumor suppressor genes *TEL* and *KIP1* in childhood acute lymphoblastic leukemia. *Cancer Res* **56**: 1413–1417

Takano H, Okamoto A, Terashima Y and Yokota J (1994) High incidence of allelic loss at the RB gene locus in advanced human ovarian cancer. *Int J Oncol* **6**: 129–135

Takeuchi S, Bartram CR, Seriu T, Miller CW, Tobler A, Janssen JWG, Reiter A, Ludwig W, Zimmermann M, Schwaller J, Lee E, Miyoshi I and Koeffler HP (1995*a*) Analysis of a family of cyclin-dependent kinase inhibitors: p15/MTS2/INK4B, p16/MTS1/INK4A and p18 genes in acute lymphoblastic ·leukemia (ALL) of childhood. *Blood* **86**: 755–760

Takeuchi S, Bartram CR, Wada M, Reiter A, Hatta Y, Seriu T, Lee E, Miller CW, Miyoshi I and Koeffler HP (1995*b*) Allelotype analysis of childhood acute lymphoblastic leukemia. *Cancer Res* **15**: 5377–5382

Takeuchi S, Bartram CR, Miller CW, Reiter A, Seriu T, Zimmerman M, Schrappe M, Mori N, Slater J, Miyoshi I and Koeffler HP (1996*a*) Acute lymphoblastic leukemia of childhood: identification of two distinct regions of deletion on the short arm of chromosome 12 in the region of *TEL* and *KIP1*. *Blood* **87**: 3368–3374

Takeuchi T, Mori N, Koike M, Slater J, Park S, Miller CW, Miyoshi I and Koeffler HP (1996*b*) Frequent loss of heterozygosity in region of the *KIP1* locus in non-small cell lung cancer: evidence for a new tumor suppressor gene on the short arm of chromosome 12. *Cancer Res* **56**: 784–740

Toyoshima H and Hunter T (1994) p27, a novel inhibitor of G1 cyclin-cdk protein kinase activity, is related to p21. *Cell* **78**: 67–74

Viel A, Giannini F, Tumiotto L, Sopracordevole F, Visentin MC and Boiocchi M (1992) Chromosomal localisation of two putative 11p oncosuppressor genes involved in human ovarian tumors. *Br J Cancer* **66**: 1030–1036

Wlodarska I, Mecucci S, Marynen P, Guo C, Franckx D, La Starza R, Aventin A, Bosly A, Martelli MF, Cassiman JJ and Van den Berghe H (1995) *TEL* gene is involved in myelodysplastic syndromes with either the typical t(5;12) (q33;p13) translocation or its variant t(10;12) (q24;p13). *Blood* **85**: 2848–2852

Xu W, Gorman P, Sher D, Bated G, Kishimoto J, Lizhi L and Emson P (1993) Regional localization of the gene coding for human brain nitric oxide synthase (NOS1) to 12q24.2–24.31 by fluorescent in situ hybridization. *Cytogenet Cell Genet* **64**: 62–63

Yang-Feng TL, Li S, Han H and Schwartz PE (1992) Frequent loss of heterozygosity on chromosome Xp and 13q in human ovarian cancer. *Int J Cancer* **52**: 575–580

Yang-Feng TL, Han H, Chen K-C, Li S, Claus EB, Carcngiu ML, Chambers SK, Chambers JT and Schwartz PE (1993) Allelic loss in ovarian cancer. *Int J Cancer* **54**: 546–551

Zheng J, Robinson WR, Ehlen T, Yu MC and Dubeau L (1991) Distinction of low-grade from high-grade human ovarian carcinomas on the basis of losses of heterozygosity on chromosome 3, 6, and 11 and *Her-2/neu* gene amplification. *Cancer Res* **51**: 4045–4051

 © Cancer Research Campaign 1997